# Metro Nature, Environmental Health, and Economic Value

**DOI:** 10.1289/ehp.1408216

**Published:** 2015-01-27

**Authors:** Kathleen L. Wolf, Alicia S.T. Robbins

**Affiliations:** College of the Environment, University of Washington, Seattle, Washington, USA

## Abstract

**Background:**

Nearly 40 years of research provides an extensive body of evidence about human health, well-being, and improved function benefits associated with experiences of nearby nature in cities.

**Objectives:**

We demonstrate the numerous opportunities for future research efforts that link metro nature, human health and well-being outcomes, and economic values.

**Methods:**

We reviewed the literature on urban nature-based health and well-being benefits. In this review, we provide a classification schematic and propose potential economic values associated with metro nature services.

**Discussion:**

Economic valuation of benefits derived from urban green systems has largely been undertaken in the fields of environmental and natural resource economics, but studies have not typically addressed health outcomes. Urban trees, parks, gardens, open spaces, and other nearby nature elements—collectively termed metro nature—generate many positive externalities that have been largely overlooked in urban economics and policy. Here, we present a range of health benefits, including benefit context and beneficiaries. Although the understanding of these benefits is not yet consistently expressed, and although it is likely that attempts to link urban ecosystem services and economic values will not include all expressions of cultural or social value, the development of new interdisciplinary approaches that integrate environmental health and economic disciplines are greatly needed.

**Conclusions:**

Metro nature provides diverse and substantial benefits to human populations in cities. In this review, we begin to address the need for development of valuation methodologies and new approaches to understanding the potential economic outcomes of these benefits.

**Citation:**

Wolf KL, Robbins AS. 2015. Metro nature, environmental health, and economic value. Environ Health Perspect 123:390–398; http://dx.doi.org/10.1289/ehp.1408216

## Introduction

More than 50% of the world’s population now live in cities and further concentration in urban areas is forecast (United Nations Population Fund 2007). Although some city governments struggle to meet basic daily needs such as safe housing, dependable utilities, and transportation, many others have achieved reliable and affordable basic systems and services. Of interest to both governments and citizens, once basic systems are in place, is the livability of urban areas and the quality of life afforded their citizens. Residents of highly urbanized centers often expect livable environments that include access to urban nature and investments in green infrastructure.

The public has long recognized that nature in cities and towns provides beauty and respite. There is now extensive evidence that both constructed and endemic nature elements can contribute significant ecosystem services (ES) that generate public health co-benefits. Services such as air and water purification, stormwater management, carbon sequestration, and reduction of heat island effects are fairly well-defined at this time (Chen and Jim 2008), and have been assessed for their potential economic values (Nowak et al. 2010). The psychosocial services provided by metro nature are of increasing interest, including the cognitive, emotional, and psychological benefits derived from interactions with nature (Bratman et al. 2012).

Consistent with the articulation of ES by the Millennium Ecosystem Assessment (2005), various programs [such as The Economics of Ecosystems and Biodiversity (TEEB) (Sukhdev et al. 2014) and Earth Economics (Schrier et al. 2013)] and systems models (e.g., Reis et al. 2013; Rounsevell et al. 2010) have addressed the complexity of macro-ecological conservation in relation to human health, including concerns of biodiversity and climate change. Embedded within these more broadly scoped ecological management pursuits are the micro-scale nature elements that can permeate the urban environment.

Micro-scale nature elements can take many forms. The term “metro nature” is used here to refer to the collective opportunities for human nature experiences that improve urban livability (Wolf 2008). The term “metropolis,” from which “metro” is derived, refers to an urbanized area made up of multiple settlements and political jurisdictions. Metro nature is a unifying concept that acknowledges cultural and ecological landscapes governed by diverse entities and landowners—both public and private—within cities. Metro nature includes endemic ecosystems, such as urban forests, greenbelts, conserved open spaces, and riparian corridors that may be patch, relic, or feral expressions of native ecological associations. It also includes culturally constructed nature such as parks, streetscapes, community gardens, pocket parks, and recreation paths. Finally, metro nature includes structural innovations that are integrated within built form to serve specific functions, such as green roofs, green walls, or green infrastructure facilities.

Recent studies have explored the definition and supply of urban ES. Papers about urban ES often represent a limited view of urban cultural aspects (Bolund and Hunhammar 1999; Gómez-Baggethun and Barton 2013; Larondelle and Haase 2013; Sander and Haight 2012) or have overlooked cultural values altogether (Jansson 2013; Li and Wu 2013). To date, the presentation and classification of urban ES does not adequately capture the full range of nature-based benefits and services within metro environments, particularly cultural ES (Wolf 2012).

The objective of this review was to demonstrate the extensive opportunities for research efforts that link metro nature, human health and well-being outcomes, and economic values. We begin by proposing a classification schematic that interprets a broader definition of ecosystem services, particularly cultural services, from an urban perspective. Methodologies for potential economic valuations of metro nature benefits are identified. We then review publications on urban nature-based benefits, summarized using the schematic. Our intent is to build on previous works that have initiated economic valuation of metro nature services and provide descriptions of a collection of human health and well-being benefits that may be readily expanded to include economic consequences. We also integrate current and future valuation opportunities.

## Metro Nature and Health

Metro nature services are provided by small-scale nearby nature in neighborhoods and communities, and may be below the consciousness of individuals. The scientific evidence of such services spans nearly 40 years and includes the contributions of diverse disciplines (Wolf 2012). An ongoing review of publications about the relationship between urban greening and human health and well-being has revealed more than a dozen themes of services and benefits, supported by > 3,000 scholarly publications (University of Washington 2014). In this review we used an iterative search process across major web search engines—such as PubMed (http://www.ncbi.nlm.nih.gov/pubmed/), JStor (http://www.jstor.org/), and Science Direct (http://www.sciencedirect.com/)—and key journals of disciplines that are active in benefits science, such as public health, environmental psychology, and natural resources. The references we collected are peer-reviewed articles that report either passive or active experiences with nearby nature and related outcomes of health and well-being. The thematic sorting was based on a content analysis of an initial collection of 300 articles.

**Figure 1:**
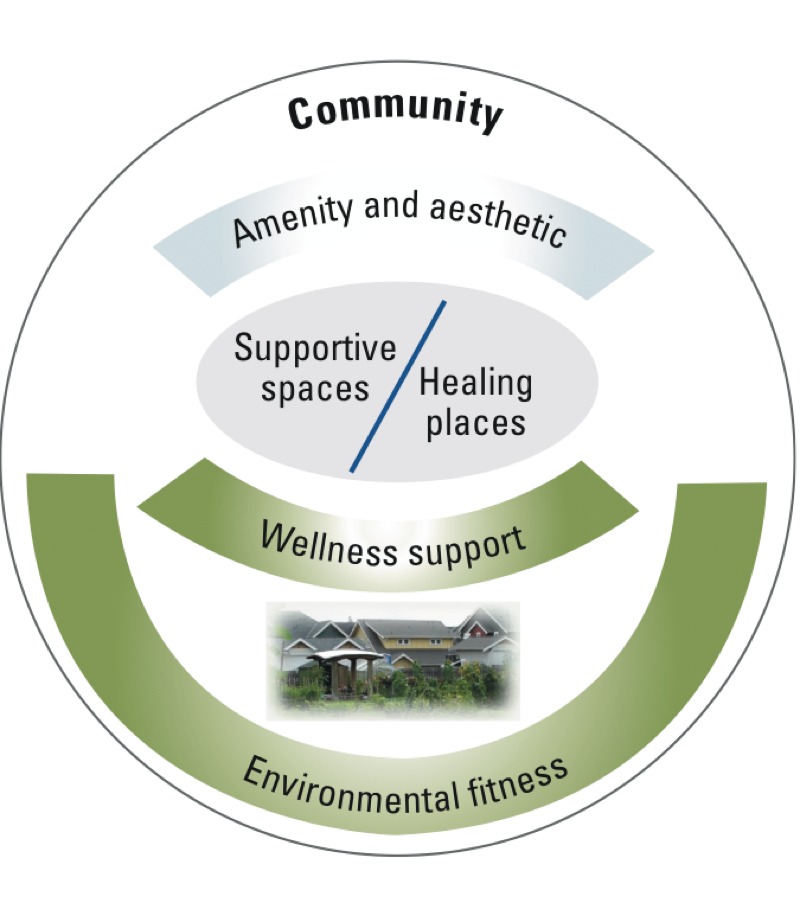
Schematic of metro nature services and benefits.

We propose a classification schematic (Figure 1) to summarize the broad array of services and benefits provided by metro nature and demonstrated in the literature described above. In this section we introduce the framework components; below we provide citations about benefits and valuation potential that expands on recent surveys of cultural values (Chan et al. 2012; Daniel et al. 2012), particularly within the context of urban environments.

“Environmental fitness” is the baseline condition of environmental support for human health. Best practices and systems of a sanitary city provide the most basic conditions necessary for good health for all city residents, such as clean air and water, and the absence of toxicants (Pincetl 2010). Environmental protection agencies at national and regional levels may monitor and regulate potential harmful impacts from pollutant emissions, harmful materials dumping, and industrial and agricultural by-products. Urban forestry and green infrastructure are increasingly utilized as prevention or mitigation strategies within both regulatory and voluntary programs of urban sustainability.

“Wellness support” represents a less fundamental, but no less important, urban condition. Recent research efforts indicate that having ubiquitous green systems such as parks, community gardens, trees, and green spaces provides supplemental benefits. Convenient and pervasive access to nearby nature includes passive views from homes and vehicles, green spaces within walkable distances, and active encounters with nature (such as gardening and tree planting); all are scientifically linked to wellness. Beneficial human responses include physical activity that can reduce incidences of chronic diseases, physiological stress moderation, and improved mental health. For instance, urban forest canopy proximate to households has been associated with higher infant birth weight (Dadvand et al. 2012) and green urban neighborhoods with reductions in elder mortality (Takano et al. 2002).

“Supportive spaces and healing places” entails more specific human responses. Common to the urban human experience are facilities and institutions where one conducts exacting routine activities (such as school or the workplace). Studies have found that nature is supportive in human performance situations as evidenced by improved workplace satisfaction (Kaplan 1993) and high school success (Matsuoka 2010). Landscape design or retention might be strategically placed to improve human function. Second, a more extensive literature has described how both passive experiences of nature and directed horticulture therapy can aid people in both physical and emotional healing. Such places include healing gardens within hospitals, horticulture therapy gardens, and sacred spaces (such as memorials). Landscapes that aid in healing or therapy are often dedicated constructed sites that include specific design elements intended to engage people for specified experiences or outcomes. In contrast, supportive spaces are expressions of nature that are adjacent to and augment places where people work, learn, or study; they provide benefits but not necessarily with the direct intention of healing places.

“Amenity and aesthetics” describes perhaps the most widely perceived benefit of trees, parks, and greening. Many in the green industries rely on client appeals of emotion and beauty, such as the Love Your Landscape messaging (Professional Landcare Network 2014). The City of Seattle, Washington (Seattle ReLeaf 2013) conducted marketing research to develop residential outreach to boost forest canopy cover; citizen responses of beauty, wonder, and spiritual connection to trees were more common than responses directed toward ecological services. Research indicates that humans respond to the presence of nature in profound ways, even after only brief exposure times, although they may not be directly aware of the outcomes. Neuroscience studies are considering the role of urban environmental influences on human wellness (Lederbogen et al. 2011).

Finally, the term “community” acknowledges that all of these experiences and associated services are embedded within the context of human systems, built places, and change. Citizens are becoming ever more involved in metro nature planning, implementation, and management. In resurgent cities, cleaning up vacant lots, restoring parks, and creating community gardens are often markers of community recovery (Harnik 2010). These acts of civic ecology can lead to social engagement and cohesion, perhaps improving local social resilience (Krasny and Tidball 2012). Studies that address neighborhoods or general human populations have suggested that nature-based activity develops social foundations that can support disaster recovery (Tidball and Krasny 2014). Additional studies point to the unequal distribution of parks and natural resources within cities and its environmental justice implications (Jennings et al. 2012; Masuda et al. 2012).

Metro nature services are potentially available to all urbanites, and any single site may provide multiple functions, as shown in Figure 1. For instance, a hospital healing garden may be used for patient-healing activities as well as a restorative setting for staff breaks. Community investment is necessary to achieve optimal levels of all such services. Yet few metro nature services have been framed in terms of their potential economic values. Defining the broadest range of economic values associated with the human health and well-being benefits of metro nature can provide decision makers and urban planners with important information when making decisions about investments in these public goods.

## Economic Methods

A number of methods are used to estimate the economic or monetary value of environmental attributes, with textbooks and journals devoted to the endeavor. The health economics field is similarly well-defined. Some approaches are used across both fields, including avoided or replacement costs, as well as decision-analysis frameworks such as benefit–cost, cost effectiveness, and cost utility. Stated preference methods were developed in environmental economics but are becoming more widely used in the medical economics literature. Only a brief overview of the methods suggested below is presented here in order to introduce concepts to readers who may be unfamiliar with their use. For more in-depth methodological explanations of these and other environmental economic methods see, for example, Champ et al. (2003) or Tietenberg and Lewis (2011); other examples have been published in the health economics literature (e.g., Culyer and Newhouse 2000; Drummond et al. 2005).

Where markets exist, prices for goods and services are used to estimate value. Factor income approaches can be used where an improvement to a resource results in an increase in incomes derived from the resource. Production functions can be estimated when resources are used as inputs to the production of goods and services and where changes to the inputs result in production changes. Cost methods include several approaches. The avoided cost method uses monetary expenditures that would be avoided by employing specific management decisions or practices.

Many ecosystem services and benefits carry no market prices, so quantifying their economic value is performed through analysis of observed or hypothetical behaviors. Hedonic pricing uses sales prices of buildings or properties to isolate the differential effect of environmental attributes on property values. The contingent valuation method, originally developed to value public goods and services, asks survey respondents to identify willingness to pay for improvements—or willingness to accept damages—to a resource (Carson 2011). Similarly, discrete choice experiments also elicit willingness to pay and willingness to accept, but can include multiple levels of attributes at different cost levels; results can be used to create a ranking of preferences for alternative conditions or scenarios. Discrete choice experiments are well-established in the environmental economics literature and are increasingly being applied in the health economics field (Carson and Louviere 2010).

Decision-making frameworks in the fields of environmental and resource economics typically employ more than one estimation method to capture all benefits and costs. Benefit–cost analysis calculates total realized and expected benefits and costs of a project or conditions over time and discounts them to net present value, with the goal of identifying the option(s) that will provide the greatest net benefit. Cost-effectiveness analysis compares the relative benefits and costs of multiple means to meet the same goal by identifying cost differentials associated with different approaches. The option that meets the objective for the least cost is selected.

Health economics methods center on the cost of illness and treatment. Many of the methods described above can be applied to disease prevention and therapies. In addition, cost-minimization methods are frequently employed. Evaluations are performed using decision-making frameworks. Value of statistical life represents the aggregation of individuals’ willingness to pay to reduce the incidence of preventable death across a population. Burden of illness methods estimate the economic burden of diseases and potential savings associated with disease eradication. Quality-adjusted life-year techniques provide a measure of the number and quality of life years added by medical treatments and disease prevention.

## Benefits and Economic Valuation: Literature to Date and Beyond

In this section we review existing literature on economic valuation of health and well-being benefits, and present benefits that have not been valued to date but warrant exploration. In these instances, benefits are defined and potential valuation methods suggested. The range of benefits is discussed in terms of the Figure 1 schematic.

*Environmental fitness*. Air pollution. Air quality improvements and CO_2_ sequestration by vegetation can be expressed in monetary terms. Nowak et al. (2006) modeled total air pollution removal by urban trees and shrubs across 55 U.S. cities at 711,000 metric tons, estimating the removal value at $3.8 billion in annual public value. Escobedo et al. (2008) quantified particulate matter removal by urban forests in Santiago, Chile, and estimated associated management costs. They compared their results with benefit estimations produced by the World Bank and found that managing urban forests to produce air quality improvements was a cost-effective approach to reducing particulate matter.

Stormwater runoff. Reduction of stormwater runoff can be achieved through planting or conserving existing forested areas and creating other green infrastructure mechanisms, such as green roofs (Mentens et al. 2006). Trees and soils improve water quality in that they can remove harmful substances washed off roads, parking lots, and roofs during rain or snow events. Vegetation can also reduce the need for costly stormwater treatment by retaining or slowing the flow of precipitation reaching the ground. These systems reduce the risk of major flooding and water treatment costs. In addition, vegetation can be planted to reduce the risk of negative effects from drought. The U.S. Environmental Protection Agency’s (EPA) National Stormwater Calculator (http://www2.epa.gov/water-research/national-stormwater-calculator) can be used to estimate annual stormwater runoff, based on site-specific information. Other tools are available from the U.S. Department of Agriculture Forest Service to calculate ecosystem services provided by an urban forest canopy (i-Tree Eco; http://www.itreetools.org/) or by street trees (i-Tree Streets; http://www.itreetools.org/streets/index.php). Analysis modules have been used to quantify multiple services (including air pollutant filtration, stormwater runoff reduction, carbon sequestration) (McPherson et al. 2005; Nowak et al. 2010; Soares et al. 2011). In some instances, monetary values have been estimated, for example, by calculating avoided or replacement costs using the difference in cost between traditional gray infrastructure installations and green infrastructure installations (Nowak and Dwyer 2007; U.S. EPA 2013).

Heat effects. One much-studied service provided by urban trees is canopy cover and shade, which in turn keeps people and buildings cooler and protected from extreme weather effects. Heat waves (and associated extreme nighttime temperatures) have been associated with detrimental health effects and excess mortality (Bowler et al. 2010). Urban forests and green roofs can aid in reducing urban heat island effects (Takebayashi and Moriyama 2007). Parks can be up to 2°F cooler than the surrounding urban area in the day (Bowler et al. 2010); large numbers of trees and expansive green spaces across a city can reduce local air temperatures by up to 9°F (McPherson 1994).

Noise abatement. There is now extensive evidence demonstrating the many negative effects of noise on health (Passchier-Vermeer and Passchier 2000). Trees and shrubs can significantly reduce noise (Fang and Ling 2003; Nowak et al. 2010). Noise and noise reduction effects on property prices have been studied, although not extensively, largely through the use of hedonic models (Day et al. 2007; Kim et al. 2007; McMillan et al. 1980). We found no studies specifically on noise abatement and economic values achieved through the use of trees, shrubs, or other green elements. Opportunities exist to use hedonic or other approaches that value aesthetic amenities. Combined approaches that use both revealed methods and choice experiments or the contingent valuation method might be also suited to this area of study.

*Wellness support*. Active living. Although studies are not consistent, research has demonstrated an association between parks and open spaces and the propensity to engage in physical activity (Ellaway et al. 2005; Giles-Corti et al. 2005). One research focus is the role home location and park proximity may play in physical activity, with mediating factors including the characteristics of routes to a park (Sugiyama et al. 2010). Schipperijn et al. (2013) reported that greater attention is now given to park attributes that promote activity. In establishing a relationship between physical activity and green space, it is important to specifically identify the type of activity undertaken (de Vries et al. 2011), site features that enable or discourage activity, frequency of green space use, and other potentially confounding factors. Links between physical activity in or near green spaces and specific health outcomes are beginning to be explored. Although findings have been mixed, outcomes include obesity reduction (Lachowycz and Jones 2011; Potwarka et al. 2008), lower blood pressure (Hartig et al. 2003), and extended life spans (Takano et al. 2002).

Economic valuations are preliminary, appearing mostly in gray literature. The Trust for Public Land (2013) calculated annual avoided costs of health care associated with levels of physical activity in parks across 10 U.S. cities and counties; values ranged from $4 million to $69.4 million per year. The Green Infrastructure Toolkit provides a calculator for estimating reduced mortality due to increased walking in new green areas in cities in the United Kingdom (Natural Economy Northwest 2011). Willis and Crabtree (2011) presented potential methods for estimating the monetary value of reduced human mortality, morbidity, and averted deaths associated with physical activity in green space; they concluded that calculating net benefits of additional green space is dependent on identifying the degree of change from sedentary to active behavior. Discrete choice experiments allow the concomitant evaluation of multiple attributes and seem particularly appropriate for research questions that require simultaneously addressing both environmental and health factors. Potential cost savings estimates could be used in cost-effectiveness or benefit–cost analyses to examine tradeoffs between building locations or enhancing green space.

Stress relief. Studies have shown that relaxation and stress reduction are associated with exposure to green views (Kahn et al. 2008; Ulrich et al. 1991) and spending time or exercising in green areas (Hansmann et al. 2007; Hartig et al. 2011), including walking in forested areas (Park et al. 2010; Tsunetsugu et al. 2010). Stress response is a contributor to short- and long-term physiological outcomes: sleep loss, suppressed immune system function, susceptibility to illness, high blood pressure, cardiovascular disease, stroke, and diabetes (National Center for Health Statistics 2001). Cost approaches could include identifying treatment reductions or alternatives that reduce burden of illness. Similarly, cost-effectiveness analysis could be employed to estimate intervention tradeoffs once treatment alternatives are identified.

Mental health. Recent studies highlight the importance of nature contact for general mental health. Aspinall et al. (2015) reported that when respondents moved into a green space zone, their electroencephalography (EEG) recorder data showed lower frustration, attentional engagement, and arousal, but higher meditation. People living in urban areas with greater amounts of green space showed significantly lower mental distress (White et al. 2013). In a study about mental health responses and green space, Annerstedt et al. (2012) found a reduced risk for poor mental health among women (but not men) through a significant interaction effect between physical activity and access to certain qualities. In a study of individuals with major depressive disorders, Berman et al. (2012) found improvements in mood associated with walking in nature. Reduced depression in the elderly after walking in gardens has been reported (Blumenthal et al. 1999; McCaffrey et al. 2010). Outdoor spaces designed for walking have also been associated with lowering symptoms of Alzheimer’s and dementia (Chalfont and Rodiek 2005; Mooney and Nicell 1992). Research is still needed to explore the potential for green spaces to supplement or be used instead of professional therapy or prescription medicine. Avoided-cost or cost-effectiveness methods might be used to estimate reductions in care costs or cost tradeoffs.

Urban food and foraging. Urban community gardens, rooftop vegetable gardens, and public orchards are examples of urban ecosystem goods production (McLain et al. 2012a). These “edible landscapes” rarely provide adequate food supplies for local urban populations (Armstrong 2000). However, they can be important sources of food to specific populations (McGranahan et al. 2005) and can support food resilience in some situations (Barthel and Isendahl 2013). In addition, recent assessments of urban gathering and foraging show that urban forests contain nontimber forest products that contribute a variety of wild foods, medicines, and materials useful for the well-being of urban residents, with some materials (such as culinary fungi) supporting household livelihoods (McLain et al. 2012b; Poe et al. 2013). Although many studies have noted the absence of adequate food sources in some inner-city areas (Walker et al. 2010), little economic valuation of urban agriculture or foraging has been done to date. One study found that the presence of community gardens had a positive impact on property values, particularly in poorer neighborhoods (Voicu and Been 2008). In areas with organized community gardens, such as those overseen by city governments, there could be opportunities to estimate production functions and value, as well as factor income effects, perhaps on a micro-community level.

Respiratory health. Findings on the links between respiratory health and vegetation or canopy cover in urban areas are mixed. Donovan et al. (2013) found a correlation between residential tree loss (due to emerald ash borer–related tree mortality) and respiratory disease. Lovasi et al. (2008) reported that street trees in New York City were associated with a lower prevalence of early childhood asthmas, but the results were questioned by Zandbergen (2009). Pilat et al. (2012) did not find statistically significant relationships between vegetation and canopy cover and childhood asthma in Texas. Further evidence on links between the presence of trees and other green elements with respect to respiratory health is needed to establish net benefits. These could then be translated into economic terms through the use of avoided-cost or cost-effectiveness methods to estimate reductions in care costs or cost tradeoffs associated with reduced respiratory illness and disease. Value of statistical life or quality-adjusted life-year methods could be used to measure the value of respiratory-related mortality risk reduction.

*Supportive spaces*. Mental function. The directed cognitive focus that is needed for task attention (in school or at work) can be improved after exposure to nature (Kaplan R 1993; Kaplan S 1995; Lohr et al. 1996, Shibata and Suzuki 2002). Studies show that improved employee morale, decreased absenteeism, and increased worker efficiency result from nature experiences while in the workplace (Lohr et al. 1996). Having plants within view of workstations decreases illness incidence (Fjeld et al. 1998) and the amount of self-reported sick leave, and boosts workplace satisfaction (Kaplan 1993). Not having nature views or indoor plants may be associated with higher levels of tension and anxiety in office workers (Chang and Chen 2005). In academic settings, nature views may lead to improved high school and college student performance (Matsuoka 2010; Tennessen and Cimprich 1995). More research is needed on individuals’ academic achievement or school rankings (e.g., test scores, graduation rates, college enrollment). Differences in graduation rates and college attendance could be associated with annual or lifetime earnings. In workplace settings, decreased absenteeism or increased productivity in the workplace could be associated with a mean or median wage level or with increased revenue or efficiency for companies.

Attention deficit disorder (ADD). Research has shown that when children are engaged in activities in green settings, childhood ADD symptoms are reduced (Taylor and Kuo 2011; Taylor et al. 2001) and concentration abilities are improved (Kuo and Taylor 2004). Spending time in green settings may be an important supplement to established drug-based and behavioral treatments (Taylor and Kuo 2009). Again, avoided-cost or cost-effectiveness methods could be used to estimate the reductions in medication and care costs or cost tradeoffs. As noted above for mental function, improved educational attainment and school performance could be linked to increased lifetime earnings.

*Healing spaces*. Physical healing. Recent research has shown that access to natural elements can aid physical healing (Sherman et al. 2005; Walch et al. 2005). Studies have reported faster surgical recovery and patient healing in hospitals (Park and Mattson 2009; Ulrich 1984) and higher pain thresholds (Diette et al. 2003; Tse et al. 2002) associated with passive nature experiences and views. One hypothesis is that nature serves as a distraction that allows individuals to refocus cognitive effort, resulting in increased pain thresholds and tolerance as well as improved coping and healing (Ulrich 1999). Another hypothesis posits that exposure to green attributes in hospitals helps to reduce cognitive stress levels (Kaplan and Kaplan 1983), which can be linked to negative health outcomes (Varni and Katz 1997). This research has prompted some hospitals to establish healing gardens (Cooper Marcus and Sachs 2013; Franklin 2012) and provide horticulture therapy programs.

Ulrich (1984) found that patients having a view of nature during surgery recovery had, on average, a 1-day shorter hospitalization stay; this is significant given the daily cost of hospital stays, which average about $1,700 in the United States (Aetna 2013). More data are needed to verify dose responses and determine how exposure to green spaces affects both in-patient and out-patient treatments, with potential reduced or avoided costs. Another method that could be applied is willingness to pay for pain-reduction treatments. Discrete choice methods could be used to survey patients with different treatment options in regard to pain levels or recovery times (Chuck et al. 2009). Quality-of-life metrics could also be used. All valuation approaches could be compared with implementation costs and used in decision-making in terms of benefit–cost tradeoffs and cost effectiveness.

Horticulture and nature therapy. Nature-based therapies typically incorporate garden activities such as design and planting, maintenance, or visitation. Therapeutic horticulture is the creation of settings and/or activities that enrich participants’ lives through interactions with the diversity of life in the natural world. Horticultural therapy is the use of an intervention or prescribed activity to address specific, diagnosed emotional and physical disabilities; activities take place in gardens and established outdoor restorative centers (American Horticultural Therapy Association 2013). In preliminary studies of gardens and nature therapy, elderly participants have reported pain reduction, improvement in attention, reduced stress, modulation of agitation, lowered need for medications and antipsychotics, and reduction of falls (Detweiler et al. 2012). Additional outcomes have included improvements for those experiencing chronic mental illness (Perrins-Margalis et al. 2000), clinical depression (Gonzalez et al. 2010), posttraumatic stress disorders (Lorber 2011), maternity care (Browning and Lee 2011), and autism (Flick 2012), and for those being served in acute health care settings (Hilbers and Satharasinghe 2013) or crisis centers (Lygum et al. 2012). Horticultural programs in prisons in the United States suggest decreased hostility (Rice and Remy 1998), reduced recidivism (Jiler 2009), and better social adjustment for juvenile offenders (McGuinn and Relf 2001). Nature therapy could be used in lieu of prescription medicine or may lead to a reduction in overnight stays or in prison costs. Lee et al. (2008) used the contingent valuation method for such valuations; other approaches might be cost effectiveness and burden of illness metrics.

*Amenities and aesthetics*. Numerous studies have estimated impacts of street trees, urban parks, and open space on property prices. Hedonic approaches are particularly common. Although studies use various specific measurements of tree cover and examine values that differ across urban locations, findings generally demonstrate a positive relationship between the proximity to green spaces, such as urban parks and forest reserves, and property prices (Anderson and West 2006; Dombrow et al. 2000; Donovan and Butry 2010; Sander et al. 2010; Thorsnes 2002). Landscape aesthetics may also positively impact spending in retail areas. Studies using stated preference methods studies have found that consumers may be inclined to spend more while shopping in districts that have quality tree canopies (Wolf 2014). In addition, there is evidence that urban forests and parks may play a significant role in attracting tourism and associated revenue (Deng et al. 2010; Majumdar et al. 2011).

*Community*. Crime and safety. Several studies have examined associations between crime and vegetation, with mixed results. Early studies focused on the perceived threats created by vegetation (Michael et al. 2001; Nasar and Fisher 1993), such as concealment and reduced sight lines. More recently, studies have shown how vegetation can contribute to reductions in domestic aggression and violent behaviors (Kuo and Sullivan 2001); assault, robbery, and burglary (Wolfe and Mennis 2012); and theft (Troy et al. 2012). The type, height, and positioning of vegetation near single-family homes may have a positive effect on nonviolent crimes such as burglary and vandalism (Donovan and Prestemon 2012). Branas et al. (2011) found that greening of vacant lots in Philadelphia, Pennsylvania, was associated with reduced gun assaults, vandalism and criminal mischief, and self-reported reductions in stress, and with increased exercise.

We found no studies that directly monetized the links between decreased crime and vegetation. The impacts of crime on property values have been widely established (Hellman and Naroff 1979; Rizzo 1979), but studies have not addressed the effects of vegetative cover and placement. Using a hedonic approach, Troy and Grove (2008) found that proximity to parks had a positive influence on property prices until crime rates reached a threshold, above which proximity to parks began to negatively influence property values. Further studies along these lines would be valuable. The value of reduced crime could also be examined as it affects community policing and law enforcement costs, as well as property insurance rates and premiums.

## Discussion

The economic valuation of benefits derived from metro nature elements has largely been undertaken in the fields of environmental and natural resource economics, but these valuations do not typically address health and well-being outcomes. Expanded research effort in the development of new interdisciplinary approaches that integrate environmental and health economics is greatly needed. Here we have presented many such opportunities.

The literature on public health economics is dominated by cost-effectiveness and cost-minimization approaches, both of which can be similarly useful for evaluating tradeoffs between public health outcomes and the costs of creating or improving urban green infrastructure. Environmental economics often addresses negative externalities that are produced as a result of human activities such as air and water pollution and overfishing. The legacy of environmental health is to address concerns of toxicants and environmental risk; equally important is the potential for wellness from benevolent nature encounters (Frumkin 2001). Urban open spaces and elements create many positive externalities that have gone largely ignored, including the benefits of active living, physical healing, and mental restoration, among others.

It is important to acknowledge limitations to these efforts. Here we focused on the positive human response to metro nature elements, but there are certain to be associated costs. There are also potential disservices to urban ecosystems, such as air pollution and diseases from animals (Gómez-Baggethun and Barton 2013). Future analysis needs to address the cumulative per capita or regional balance of services to disservices. There may be overlapping benefits and interdependencies among benefits, discrepancies between payee and beneficiary, and inter-temporal issues. Our intent was to identify a range of benefits; it is likely that ecosystem functions overlap. Finally, because investigations of urban benefits are in the early stages, it is likely that any attempt to link ES and economic values will necessarily be incomplete.

Although there is already considerable research demonstrating positive links between metro nature and public health, Frumkin (2012) pointed out that additional study is needed and many questions are still unanswered. For instance, what are the mechanisms through which nature contact improves health and well-being, how should it be delivered, and at what dose and for how long? Forthcoming studies will address these questions, but the science is nascent. Additional questions include the spatial and temporal dimensions of nature experience and response. Identifying the natural resource elements and relevant populations of benefit is paramount. This may be key to identifying both service and valuation potentials because land use designations (e.g., residential vs. institutional) can be indicative of potential populations and service. Furthermore, landscape treatment is an important consideration. Some benefits appear to be generated by the mere presence of tree canopy; others are dependent on the presence of more detailed and refined landscape treatments at greater cost.

## Challenges

Empirical assessment of how urban forests and greenery affect health outcomes and quality of life poses analytical challenges because pathways linking the two are complex (Lachowycz and Jones 2013). There are direct effects where closeness to nature has intrinsic healing effects. Innate responses may be due to neuroanatomy (Kim et al. 2010), endocrine response (such as cortisol reduction), or para/sympathetic nerve system activity (Park et al. 2010). In contrast, some pathways include mediating conditions where urban greening either changes an exposure (such as air pollution) or behaviors (such as active use of trails) that lead to beneficial health outcomes. Measuring these contingencies involves pooling expertise from multiple disciplines, as well as assuring that all variables are commensurate in scale. Cross-sectional studies have limited applicability in drawing causal inferences between nature situations and health outcomes. Given that performing randomized control trials with urban nature interventions and health are practically infeasible (and perhaps unethical), statistical techniques (e.g., propensity-score matching) using natural experiments, as well as carefully designed case–control quasi-experimental studies are necessary to increase the evidence base on this issue.

## Conclusions

Nearly 40 years of research provides a body of evidence about benefits of human health, well-being, and improved function associated with experiences of nearby nature in cities. Yet research methods and measures are diverse in concept and implementation, presenting important concerns and challenges for monetary translation.

Although it is not necessary to frame all health and well-being outcomes in monetary terms, doing so is often effective at capturing both the public’s attention as well as that of governmental leaders and policy makers. Considering the importance of valuation in public policy and decision making, there may be value in developing a platform of common assessment that standardizes benefit measurement and nature units. Future research on benefits could then generate comparable findings as values for policy inputs across communities and metro areas.

Based on previous research, there is a clear need for development of valuation methodologies and new approaches to understanding the potential economic outcomes of these benefits. Many urban ES can be effectively provided to serve multiple public needs. When it can be shown that they have a true impact on health and quality of life, society may begin to appreciate and act on their full value.
